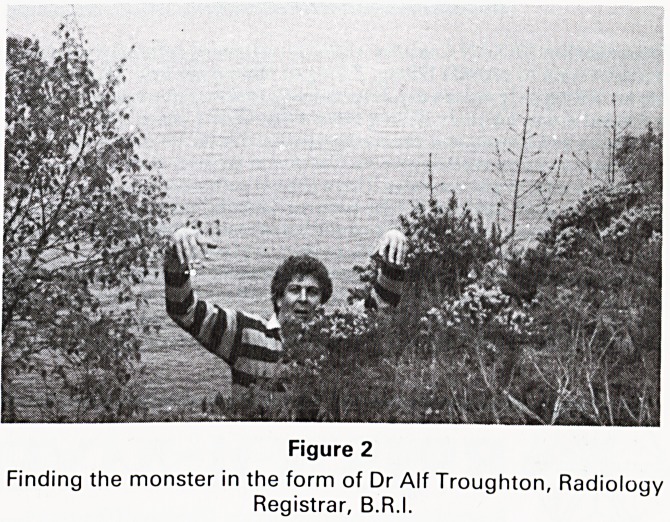# On a Bicycle Made for Two

**Published:** 1986-08

**Authors:** Alf Troughton, Rick Taylor

**Affiliations:** Registrar Radiology, Bristol Royal Infirmary; General Practitioner


### On a Bicycle Made for Two

**Alf Troughton**
Registrar Radiology, Bristol Royal Infirmary
**Rick Taylor**
General Practitioner

Swooping down the hill on our pre-war tandem bicycle, with the sun on our backs and the sea glittering in the haze below we felt pleased with ourselves. It was only lunchtime yet we had already covered over thirty miles through some of Scotland's most beautiful but mountainous terrain. With the imposing Cuillin hills stretching up on our left, their heads in the clouds, we felt much the same as we noticed a hotel in the valley below us.

Half a mile earlier at the top of the hill summoning all our flagging strength, we had just managed to catch up with a couple riding modern touring bicyles. Twelve gears through which they could slip in a moment—what a luxury that must have been. None of the torrents of abuse and painful perinei that we suffered lurching from first gear to fifth and back again. Gravity assisted, we were now a force to be reckoned with, as our combined weight of 420lbs plus heavy-weight iron machine caused us to hurtle past them. Stifling our wheezing and gasping we nonchalantly sounded off both horn and bell in derisory salute and left them floundering in our wake. The husband was last heard complaining to his wife about the amount of gear she had brought along and then made him carry on his bicycle.

We negotiated the turn into the rather smart hotel car park with accustomed difficulty, the three brakes tugging ineffectually on the wheel rims and our feet scuffing the gravel until we eventually wobbled to a halt. In matching army issue shorts, and rugby shirts and with our in flight entertainment of Sony Walkman and twin head-sets dangling from the front wicker basket, we felt slightly out of place.

Standing by the hotel wall was an anorexic looking fitness fanatic in full racing kit, doing minor adjustments to his bicycle. It was an up to the minute streamlined model that probably cost more to buy than most motorcycles and some motors cars. The most dramatic feature of it however was a perspex windshield wrapped around the front, rather like those seen on Vespa motor-scooters, but much more aerodynamic.

We looked at this piece of modern technology in wonder, and then at our own elderly packhorse. 'Er, does it make the bike go faster?' we asked. He looked at us with pity for asking such a stupid question.

'The idea is to keep the flies off when travelling at speed' he said.

I think we lost a friend as we replied 'Oh, we take regular baths, it seems to have the same effect.'

Swaggering off as best we could, with sore glutei and impending chondro-malacia patellae, we headed into the bar for a couple of well earned lager shandies.

**Figure 1**
Dr Richard Taylor, GP Shirehampton, looking for the Loch Ness Monster.

**Figure 2**
Finding the monster in the form of Dr Alf Troughton, Radiology Registrar, B.R.I.

## Figures and Tables

**Figure 1 f1:**
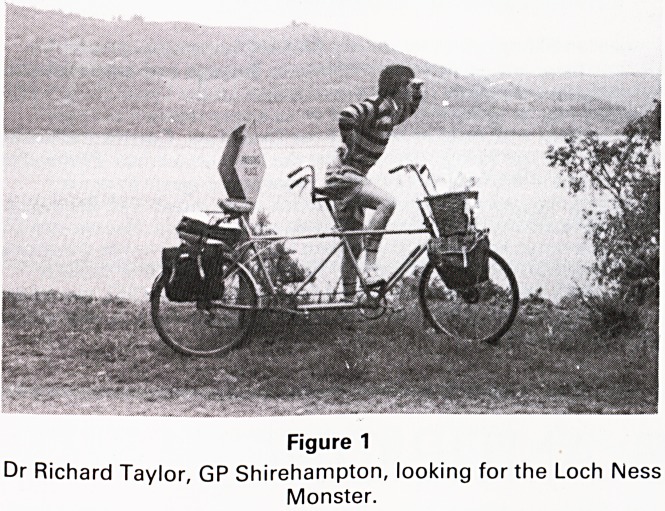


**Figure 2 f2:**